# The microbiological spectrum and clinical course of adolescents and adults with peritonsillar abscesses

**DOI:** 10.1038/s41598-026-61561-z

**Published:** 2026-08-04

**Authors:** Julia van de Loo, Maya Kurzawa, Alessa Boschert, Louis Jansen, Philipp Wolber, Lisa Nachtsheim, Hans Eckel, Kariem Sharaf, Maria Ziogas, Anne Nobis, Charlotte Klasen, Malte Suchan, Jens Peter Klussmann, Marcel Mayer

**Affiliations:** 1 Department of Otorhinolaryngology, Head and Neck Surgery, Medical Faculty, University of Cologne, 50924 Cologne, Germany; 2 Institute for Medical Microbiology, Immunology and Hygiene, Medical Faculty, University of Cologne, Cologne, Germany; 3 MVZ Laboratory Dr. Limbach & Colleagues eGbR, Heidelberg, Germany

**Keywords:** Peritonsillar abscess, *Fusobacterium necrophorum*, Lemierre’s syndrome, Pathogenicity, Antibiotics, Treatment strategies, Microbiology, Medical research, Outcomes research, Paediatric research

## Abstract

A peritonsillar abscess (PTA) displays a purulent infection arising from acute tonsillitis, involving a variety of aerobic and anaerobic bacteria. *Fusobacterium necrophorum*, a gram-negative anaerobe, is considered a significant risk factor for complications like Lemierre’s syndrome. This study aimed to analyze the microbiological spectrum of PTA and its impact on the clinical course and outcomes. This retrospective study included all patients diagnosed with PTA at a tertiary care center between January 2018 and June 2023. Data on patient demographics, inflammatory markers, microbiological aspects (including species identification and antibiotic therapy), and clinical management were collected. Overall, 321 patients with a mean age of 34.1 years were included. Most patients (80.1%) underwent tonsillectomy, while others received surgical incision and drainage (18.1%) or no surgical treatment (1.9%). Patients with tonsillectomy had a significantly longer hospital stay compared to those with incision and drainage (5.4 ± 2.5 days vs. 4.4 ± 1.3 days; adjusted *p* = 0.004). Surgical complications were more frequent in patients with tonsillectomy (13.6% vs. 1.7%; adjusted *p* = 0.048), whereas ipsilateral recurrence was more common in patients with incision and drainage (8.6% vs. 0.0%; adjusted *p* = 0.004). Higher CRP levels at admission (*p* = 0.002) were predictive for a higher risk of infection-related complications. *Streptococcus pyogenes* was the most frequently identified pathogen (25.9%), followed by *Streptococci* of the anginosus group (15.3%), and *Fusobacterium necrophorum (FN;* 11.5%), which was significantly more common in adolescents (age < 18 years; 25.3%) than in adult patients (9.5%; adjusted *p* = 0.020) and had high antibiotic susceptibility rates with 100% to clindamycin and 96.6% to penicillin or aminopenicillin with beta-lactamase inhibitor, the latter being administered to the majority (87.2%) of patients as empiric antibiotic treatment. This study provides insights into the demographic, clinical, and microbiological characteristics of PTAs. Consistent with previous literature, this study shows a higher frequency of *FN* in PTAs in adolescents and young adults. However, unlike previous reports, this was not associated with increased complication rates.

## Introduction

A peritonsillar abscess (PTA) is a common deep neck infection that typically arises as a complication of acute tonsillitis. It is characterized by the accumulation of pus between the tonsillar capsule and the superior constrictor muscle^1^. PTA is a polymicrobial infection involving a diverse range of aerobic and anaerobic bacteria, with *Streptococcus pyogenes*, *Fusobacterium necrophorum (FN)*, and *Prevotella species* being among the most commonly described pathogens^2–4^. Viral pathogens are not typically associated with abscess formation^5–7^. Known risk factors for PTA include poor oral hygiene, immune deficiency and nicotine abuse^8,9^. Therapeutic management consists of systemic antibiotics and interventional procedures like tonsillectomy or local incision and drainage. In addition, needle aspiration is a recognized option for source control and to guide targeted antimicrobial therapy in PTA. Interventional therapy can be performed under local or general anesthesia depending on patient-specific risk factors (e.g., advanced age, multimorbidity) and the hospital’s standard protocol^1,10^. While most cases resolve with appropriate antibiotic therapy and surgical drainage, a small percentage can progress to the life-threatening condition such as Lemierre’s syndrome. Lemierre’s syndrome, also referred to as post-anginal septicemia or necrobacillosis, is a rare but potentially fatal complication of oropharyngeal infections. It is characterized by thrombophlebitis of the internal jugular vein and septic emboli, most commonly affecting the lungs. The primary causative agent is the anaerobic gram-negative rod *FN* which is part of the normal oropharyngeal flora and can also be seen with asymptomatic pharyngeal carriage^11,12^. The first description of Lemierre’s syndrome dates back to Lemierre (1936), where PTA was often part of the presentation; in contrast, later studies show this combination is now rare, likely due to early antibiotic treatment^13^. However, recent population-based studies indicate a renewed increase in invasive infections and Lemierre’s syndrome, potentially due to changes in antibiotic prescribing practices^14^. Early recognition and prompt treatment with appropriate antibiotics and supportive care are crucial to prevent potentially life-threatening complications^13,15–17^. Previous studies have shown an age-dependent distribution of *FN*, with the highest prevalence in adolescents and young adults. However, little is known about younger pediatric patients, as prior cohorts largely excluded this group. Recent studies have reported *FN* in both symptomatic and asymptomatic oropharyngeal carriage among adolescents^11,12^, supporting its clinical relevance across age groups.

While there are several studies detailing the surgical therapy for PTA, there remains insufficient evidence regarding the optimal treatment and comprehensive analysis of microbiological data. Understanding the microbial spectrum is crucial for guiding appropriate antibiotic therapy and preventing potential complications. Therefore, this study aimed to elucidate the microbiological spectrum and its association with clinical characteristics, complications including recurrence in PTA, with reference to recent findings on a recently reported increase of complications associated with *FN*^14,18^. Moreover, we aimed to investigate differences in the clinical course following different surgical treatment approaches.

## Methods

**Fig. 1:**
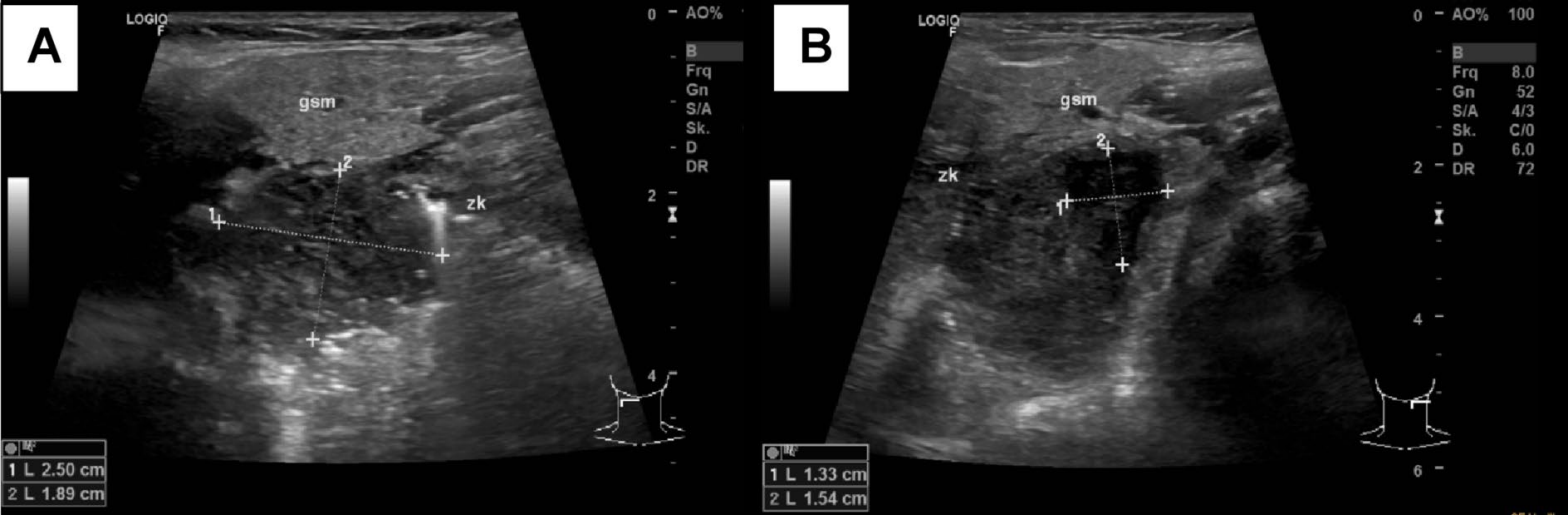
Transverse sonographic images showing (**A**) an enlarged heterogeneous right-sided tonsil without signs of an abscess and (**B**) an enlarged heterogeneous left-sided tonsil with a peritonsillar hypoechoic, partly anechoic mass (1.33 × 1.54 cm) displaying a peritonsillar abscess in a 27-year-old female patient; zk: tongue; gsm: submandibular gland.

The study included all patients diagnosed with PTA at the Department of Otolaryngology, Head and Neck Surgery of the University Hospital Cologne between January 2018 and June 2023. Diagnoses were based on clinical examination including sonography of the head and neck (Fig. 1), and CT scans of the head and neck were performed in case of clinically inconclusive findings. PTA diagnosis was confirmed by the presence of pus during tonsillectomy, incision, or aspiration. The final diagnosis of PTA was made when pus was present during tonsillectomy, incision and drainage, or when CT scans where suggestive for PTA in the limited number of cases without surgical treatment. Data on demographics, microbiological aspects, and clinical management were collected from medical records. At our institution, all patients diagnosed with PTA are admitted for inpatient management, reflecting local clinical policy. Patient demographics included sex, age, predisposing factors such as nicotine abuse, and comorbid diseases. Additionally, inflammatory markers were recorded at both admission and discharge.

Microbiological data included species identification, pathogenicity, types of antibiotic therapy, adjustments of antibiotics, antibiotic sensitivity testing, and smear test results. Clinical specimens obtained intraoperatively were incubated for seven days according to standard laboratory protocols. Species were identified using matrix-assisted laser desorption/ionization time-of-flight mass spectrometry (MALDI-TOF MS; Bruker Daltonic GmbH, Bremen, Germany), and phenotypic resistance was primarily determined using Vitek2^®^ (bioMérieux, Nürtingen, Germany), or agar disc diffusion according to EUCAST standards when necessary. Results were categorized based on the pathogenicity of detected species and their combinations into “relevant”, “possibly relevant,” and “probably not relevant.” Relevant species included *Streptococci*, *Staphylococcus aureus*, *Fusobacterium necrophorum*, and multiple strict anaerobes. Specimens with up to three potentially, albeit low virulent species of the normal oropharyngeal flora were deemed as possibly relevant. In addition, anaerobic mixed flora with no other pathogen present was also considered to be possibly relevant. Polymicrobial specimens containing only physiological aerobic oral flora were categorized as not relevant. Complications were defined as life-threatening or clinically serious events plausibly related to PTA and were divided into surgical and infection-related complications. Surgical complications included postoperative bleeding and admission to the intensive care unit (ICU) due to severe postoperative swelling. Postoperative bleeding was defined as any bleeding requiring active medical or surgical intervention (re-cauterization, suture, transfusion, or readmission). Infection-related complications included recurrence, spread of infection to adjacent structures, Lemierre’s syndrome and death. Surgical and infection-related complications were analyzed separately. Clinical management data included the length of hospital stay, duration of antibiotic administration, surgical drainage techniques, recurrence rates, and complications. Differences regarding the clinical course of patients following tonsillectomy versus incision and drainage were investigated. We compared patients with detection of each of the four major pathogens (*F. necrophorum*,* S. pyogenes*,* S. anginosus* and *S. dysgalactiae*) individually with patients in whom none of these four pathogens were detected with regard to the outcome parameters. The study adhered to the Declaration of Helsinki and was approved by the Ethics Committee of the University of Cologne (IRB approval code: 24-1365-retro). Informed consent was waived by the Ethics Committee of the University of Cologne due to the retrospective design of the study. Statistical analyses were conducted using IBM SPSS Statistics for Mac version 29.0.1.0 (171) (IBM, Armonk, NY) and R version 4.4.1 in RStudio version 2024.4.2.764. Categorical data were presented as percentages, while continuous data were expressed as means ± standard deviation (SD). The student’s t-test was used to compare the means of continuous variables between two independent groups, and the chi-squared test was used to compare the frequencies of categorical variables between two groups. To control for multiple comparisons, p-values were adjusted using the Benjamini-Hochberg false discovery rate procedure and adjusted p-values were reported^19^. One-way ANOVA was used to compare the means of continuous variables between more than two independent groups and Tukey’s test was used to correct for multiple testing. Univariate and multiple binary logistic regression analyses were performed to assess the influence of C-reactive protein (CRP) and leucocyte count on the occurrence of postoperative complications. P-values < 0.05 were considered statistically significant.

## Results

### Patients’ characteristics

A total of 321 patients with peritonsillar abscesses, of whom 138 (43.0%) were female, were included. The mean age was 34.1 years (± 17.3). Among the non-adult patients, the mean age was 10.8 ± 4.7 years. Of those, 18 were female (47.4%) and 20 were male (52.6%), 17 patients (44.7%) were adolescents (14–17 years) and five patients (13.2%) were younger than 6 years. Abscesses were located only on the right side in 134 cases (41.7%), only on the left side in 159 cases (49.5%), and bilaterally in 28 cases (8.7%; Table 1).

**Table 1:** Patients’ and procedural characteristics.

	*n* (%) or mean ± SD
Total population	321 (100.0)
Sex (female)	138 (43.0)
Mean age (years)	34.1 ± 17.3
Location right side	134 (41.7)
Location left side	159 (49.5)
Location bilateral	28 (8.7)
CRP at admission (mg/l)	107.7 ± 84.4
Leukocytes at admission (x10^3^/µl)	14.6 ± 4.9
Leukocytes at discharge (x10^3^/µl)	7.8 ± 2.9
Risk factors	
Diabetes mellitus^&^	7 (2.2)
Other immunosupression^§^	8 (2.5)
Cigarette smoking	50 (15.6)
Type of procedure	
Tonsillectomy	257 (80.1)
Incision and drainage	58 (18.1)
None	6 (1.9)
Type of anaesthesia	
General anaesthesia	260 (81.0)
Local anaesthesia	55 (17.1)
None	6 (1.9)
Surgical complications	36 (11.2)
Postoperative bleeding	28 (8.7)
ICU-admission postoperatively due to swelling	8 (2.5)
Infection-related complications	15 (4.7)
Recurrence	8 (2.5)
Ipsilateral recurrence	5 (1.6)
Contralateral recurrence	3 (0.9)
Parapharyngeal abscess	5 (1.6)
Necrotizing fasciitis + mediastinitis	1 (0.3)
Death due to septic shock	1 (0.3)
Lemierre‘s syndrome	0 (0.0)

SD: standard deviation; CRP: C-reactive protein.

^§^Lymphoma in 3 cases, immunosuppressive chronic inflammatory bowel disease in three cases, human immunodeficiency virus in one case, post-liver-transplant in one case.

^&^Type II in six cases and pancreoprive in one case; ICU: intensive care unit.

The average hospital stay was 5.2 ± 2.3 days, with no significant difference between children/adolescents and adults (4.9 ± 1.7 vs. 5.2 ± 2.4, adjusted *p* = 0.670). The mean CRP level at admission was 107.7 ± 84.4 mg/l, with higher levels in adults compared to children/adolescents (111.5 ± 85.8 vs. 79.4 ± 67.4, adjusted *p* = 0.150). The mean leukocyte count at admission was 14.6 ± 4.9 × 10^3^/µl, showing no significant difference between adults and children/adolescents (adjusted *p* = 0.400). At discharge, the mean leukocyte count was 7.8 ± 2.9 × 10^3^/µl. Seven patients (2.2%) had diabetes mellitus, and eight (2.5%) had other forms of predisposing immunosuppression (three with lymphoma, three with chronic inflammatory bowel disease under immunosuppression, one patient with HIV and one post-liver transplant). Additionally, 50 patients (15.6%) were smokers at the time of admission.

### Surgery and complications

The majority of patients underwent tonsillectomy (257 cases, 80.1%), while 58 patients were treated with surgical incision and drainage (18.1%), and 6 patients were not treated surgically (1.9%). Overall, 51 patients (15.9%) experienced complications. Surgical complications occurred in 36 (11.2%) patients with postoperative bleeding in 28 patients (8.7%) and admission to the intensive care unit (ICU) due to severe postoperative swelling in eight patients (2.5%). 15 patients (4.7%) developed infection related complications: Parapharyngeal abscesses developed in five patients (1.6%), one patient developed necrotizing fasciitis and mediastinitis and one patient had a septic shock resulting in death. Recurrence occurred in eight patients (2.5%), with five (1.6%) being ipsilateral and three (0.9%) contralateral. In one patient an Epstein–Barr virus (EBV) infection was detected after the operation, which was not classified as a complication due to PTA. No cases of Lemierre’s syndrome were observed.

Patients who underwent tonsillectomy had significantly longer hospital stays (5.4 ± 2.5 days vs. 4.4 ± 1.3 days, adjusted *p* = 0.004) and a higher risk of surgical complications (*n* = 35 (13.6%) vs. 1 (1.7%), adjusted *p* = 0.048). Patients who underwent incision and drainage had a significantly higher risk of ipsilateral recurrence (*n* = 5 (8.6%) vs. 0 (0.0%), adjusted *p* = 0.004), but there was no difference in overall infection-related complications (Tonsillectomy *n* = 10 (3.9%) vs. 5 (8.6%), adjusted *p* = 0.336, Table 2).

**Table 2:** Statistical association between microbiological spectrum, surgical therapy, age and clinical variables.

Variable (days, mean ± SD or *n* (%))	*F. necrophorum* (FN) present *n* = 37 (11.5%)	Absence of FN, GAS, SAG, SDSE *n* = 163 (50.8%)	*p*	Adjusted *p*
Hospital stay	5.1 ± 1.5	5.2 ± 1.6	0.740	0.888
CRP at admission (mg/l)	127.2 ± 96.9	97.5 ± 83.4	0.065	0.390
Leukocytes at admission (10^3^/µl)	14.7 ± 5.7	13.8 ± 5.1	0.355	0.573
Surgical complications	6 (16.2)	18 (11.0)	0.382	0.573
Infection-related complications	2 (5.4)	8 (4.9)	0.900	0.900
Recurrence	2 (5.4)	4 (2.5)	0.342	0.573

Variable (days, mean ± SD or n (%))	*S. pyogenes (GAS)* present 83 (25.9)	Absence of FN, GAS, SAG, SDSE *n* = 163 (50.8%)	p	Adjusted p
Hospital stay	4.8 ± 1.3	5.2 ± 1.6	0.088	0.264
CRP at admission (mg/l)	102.7 ± 69.7	97.5 ± 83.4	0.624	0.624
Leukocytes at admission (10^3^/µl)	15.6 ± 4.2	13.8 ± 5.1	0.009*	0.054
Surgical complications	7 (8.4)	18 (11.0)	0.522	0.624
Infection-related complications	1 (1.2)	8 (4.9)	0.144	0.288
Recurrence	1 (1.2)	4 (2.5)	0.512	0.624

Variable (days, mean ± SD or n (%))	*S. anginosus group* (SAG) present *n* = 49 (15.3)	Absence of FN, GAS, SAG, SDSE *n* = 163 (50.8%)	p	Adjusted p
Hospital stay	5.8 ± 4.7	5.2 ± 1.6	0.333	0.500
CRP at admission (mg/l)	141.7 ± 98.7	97.5 ± 83.4	0.002*	0.012*
Leukocytes at admission (10^3^/µl)	15.7 ± 4.2	13.8 ± 5.1	0.023*	0.070
Surgical complications	6 (12.2)	18 (11.0)	0.816	0.816
Infection-related complications	5 (10.2)	8 (4.9)	0.175	0.350
Recurrence	2 (4.1)	4 (2.5)	0.530	0.636

Variable (days, mean ± SD or n (%))	*S. dysgalactiae* (SDSE) present *n* = 7 (2.2)	Absence of FN, GAS, SAG, SDSE *n* = 163 (50.8%)	p	Adjusted p
Hospital stay	4.6 ± 0.8	5.2 ± 1.6	0.332	0.422
CRP at admission (mg/l)	141.9 ± 106.6	97.5 ± 83.4	0.175	0.422
Leukocytes at admission (10^3^/µl)	13.6 ± 2.7	13.8 ± 5.1	0.890	0.890
Surgical complications	0 (0.0)	18 (11.0)	0.352	0.422
Infection-related complications	1 (14.3)	8 (4.9)	0.278	0.422
Recurrence	1 (14.3)	4 (2.5)	0.070	0.420

Variable (days, mean ± SD or n (%))	Tonsillectomy *n* = 257 (80.1%)	Incision and drainage *n* = 58 (18.1%)	p	Adjusted p
Hospital stay	5.4 ± 2.5	4.4 ± 1.3	< 0.001*	0.004*
CRP at admission (mg/l)	108.1 ± 81.1	108.1 ± 93.2	0.996	0.996
Leukocytes at admission (10^3^/µl)	14.9 ± 4.8	13.6 ± 5.0	0.084	0.134
Surgical complications	35 (13.6)	1 (1.7)	0.024*	0.048*
Infection-related complications	10 (3.9)	5 (8.6)	0.252	0.336
Recurrence	3 (1.2)	5 (8.6)	0.004*	0.011*
Ipsilateral recurrence	0 (0.0)	5 (8.6)	< 0.001*	0.004*
Contralateral recurrence	3 (1.2)	0 (0.0)	0.686	0.784

SD: standard deviation; CRP: C-reactive protein; F.: Fusobacterium; FN: Fusobacterium necrophorum; GAS: Group A streptococci; SAG: Streptococcus anginosus group; SDSE: Streprococcus dysgalactiae subspecies equisimilis; S.: Streptococcus; S. anginosus group contains S.anginosus, S. intermedius and S. constellatus; significant p values in bold font.

Univariate logistic regression showed that higher CRP levels were predictive for a higher risk of infection-related complications (*p* < 0.001, OR = 0.000, 95% CI = 0.000-0.001) but not surgical complications (*p* = 0.596). Higher leucocyte counts were not associated with either type of complication (*p* = 0.534, *p* = 0.056). Multiple logistic regression proved neither CRP levels nor leucocyte count as a predictor for a higher risk of surgical complications (*p* = 0.809, *p* = 0.850) after adjustment for age, presence of *FN*, *S. pyogenes*, *S. anginosus* or *S. dysgalactiae* and the type of treatment. Higher CRP levels remained an independent risk factor for infection-related complications in the multivariable regression analysis, although the effect size was not clinically meaningful (*p* = 0.002, OR = 0.000, 95% CI = 0.000-0.001).

### Microbiological analysis and antibiotics

Most patients (*n* = 280, 87.2%) received an aminopenicillin +/- beta-lactamase inhibitor as empiric antibiotic therapy, followed by clindamycin (*n* = 30; 9.3%). The mean overall duration of antibiotic therapy (intravenous + oral administration) was 8.5 ± 2.7 days, the mean duration of initial intravenous administration was 5.2 ± 2.3 days (Table 3). In only ten patients (3.1%) the empirically used antibiotic agent was changed according to the antibiogram or escalated due to the clinical course. An antibiogram was available in 195 cases (60.7%). In 28 of the 37 patients with *FN* detected in the abscess material, antimicrobial susceptibility testing was available. The resistance profile of the organism was as follows: resistance to penicillin and ampicillin/sulbactam was observed in only one case (3.6%); however, this isolate remained susceptible to clindamycin. The remaining 27 isolates (96.4%) were susceptible to both antibiotics. All *FN* isolates demonstrated susceptibility to clindamycin (100%). Resistance to meropenem was found in two cases (7.1%), and resistance to metronidazole in one case (3.6%), while overall susceptibility to all other tested antibiotics was retained in these cases.

**Table 3:** Antibiotic therapy.

	*n* (%) or mean ± SD
Duration of i.v. (days)	5.2 ± 2.3
Duration of p.o. (days)	4.3 ± 1.2
Overall duration (days)	8.5 ± 2.7
Primarily used Antibiotic agent(s)	
Ampicillin/Sulbactam	279 (86.9)
Ampicillin/Sulbactam + Clindamycin	1 (0.3)
Clindamycin	29 (9.0)
Penicillin G	4 (1.2)
Penicillin G + Linezolid	1 (0.3)
Amoxicillin/Clavulanic acid	1 (0.3)
Cefuroxim	1 (0.3)
Cefuroxim + Metronidazol	1 (0.3)
Piperacillin/Tazobactam	1 (0.3)
Meropenem	1 (0.3)
Meropenem + Clindamycin	1 (0.3)
Meropenem + Vancomycin + Clindamycin	1 (0.3)
Change of antibiotic necessary	10 (3.1)

SD: standard deviation; i.v.: intravenous; p.o.: per os.

**Fig. 2:**
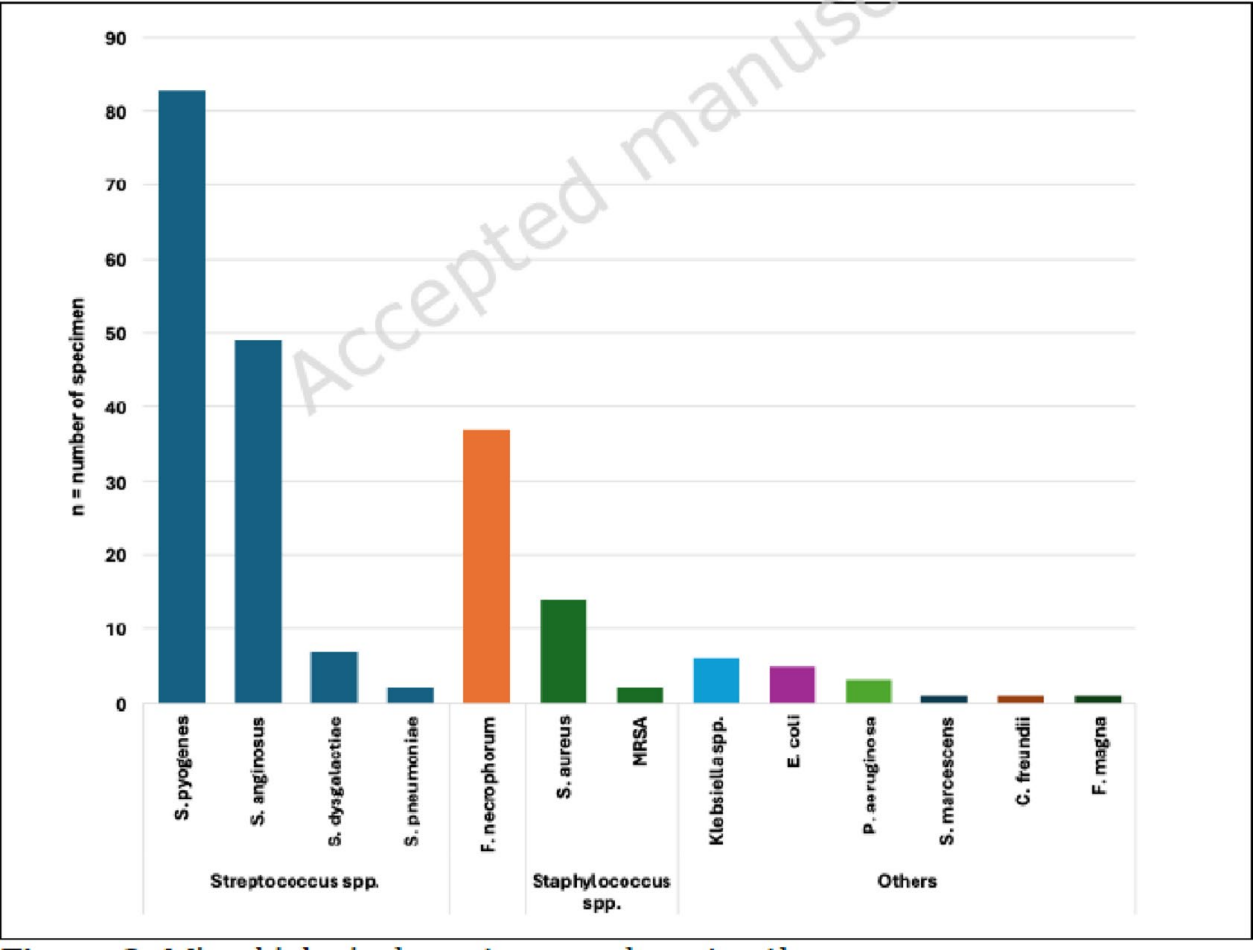
Microbiological spectrum—relevant pathogens. spp: species; F.: Fusobacterium; E.: Escherichia; P.: Pseudomonas; S.: Serratia; C.: Citrobacter; F.: Finegoldia.

Figure 2 displays the microbiological spectrum of identified relevant pathogens. The most frequently identified relevant organism was *Streptococcus pyogenes* (*n* = 83, 25.9%), followed by *Streptococci* of the anginosus group (*n* = 49, 15.3%), and *Fusobacterium necrophorum* (*n* = 37, 11.5%). In children/adolescents, *Fusobacterium necrophorum* occurred significantly more frequently compared to adults (26.3% vs. 9.5%, adjusted *p* = 0.020). Of the 37 patients with *FN*, the mean age was lower compared to patients with other pathogens (25.4 ± 12.5 years vs. 35.2 ± 17.6 years, *p* < 0.001) and 40.5% were female (*n* = 15). Among children/adolescents in whom *FN* was detected in the abscess (*n* = 10; 26.3% of the population), the majority were adolescents aged 14–17 years (*n* = 9; 90.0%). Staphylococcus aureus was identified in 16 cases (5.0%), including two cases of methicillin-resistant Staphylococcus aureus (MRSA). Moreover, several gram-negative anaerobic bacteria where found, including five cases of *Escherichia coli*, six cases of *Klebsiella spp*. and three cases of *Pseudomoas aeruginosa (*Table 4*)*.

**Table 4:** Microbiological spectrum—relevant bacteria.

	*n* (%)
*Streptococcus pyogenes*	83 (25.9)
*Streptococcus anginosus group*	49 (15.3)
*Streptococcus dysgalactiae*	7 (2.2)
*Streptococcus pneumoniae*	2 (0.6)
*Fusobacterium necrophorum*	37 (11.5)
*Staphylococcus aureus*	14 (4.4)
*Methicillin-resistant S. aureus*	2 (0.6)
*Escherichia coli*	5 (1.6)
*Klebsiella pneumoniae*	2 (0.6)
*Klebsiella oxytoca*	2 (0.6)
*Klebsiella varicola*	1 (0.3)
*Klebsiella aerogenes*	1 (0.3)
*Pseudomonas aeruginosa*	3 (0.9)
*Serratia marcescens*	1 (0.3)
*Citrobacter freundii*	1 (0.3)
*Finegoldia magna*	1 (0.3)

S.: Staphylococcus.

Among the examined pathogen specimens, 116 cases (36.1%) contained a gram-negative mixed infection with various species. *Prevotella spp.* (*n* = 45), *Veillonella spp.* (*n* = 28) and *Parvimonas micra* (*n* = 15) were the most frequent possibly relevant bacteria. *Candida spp.* was found in ten cases (Table 5).

**Table 5:** Microbiological spectrum—possibly relevant and probably not relevant organisms.

	*n* (%)
Possibly relevant	
Anaerobic mixed flora	29 (9.0)
*Prevotella spp.*	45 (14.0)
*Veillonella spp.*	28 (8.7)
*Parvimonas micra*	15 (4.7)
*Candida spp.*	10 (3.1)
*Actinomyces odontolyticus*	6 (1.9)
*Fusobacterium nucleatum*	6 (1.9)
*Eggerthia catenaformis*	4 (1.2)
*Campylobacter spp.*	4 (1.2)
*Atopubium spp.*	3 (0.9)
*Bacteroides fragilis*	2 (0.6)
*Peptostretococcus anaerobius*	2 (0.6)
*Actinomyces graevenitzii*	2 (0.6)
*Staphylococcus epidermidis*	1 (0.3)
*Fusobacterium gonidiaformans*	1 (0.3)
*Dialister microaerophilius*	1 (0.3)
*Eikenella corrodens*	1 (0.3)
*Haemophilus influenzae*	1 (0.3)
Probably not relevant	
Aerobic mixed flora	53 (16.5)
*Selenomonas infelix*	1 (0.3)

spp: species.

**Fig. 3:**
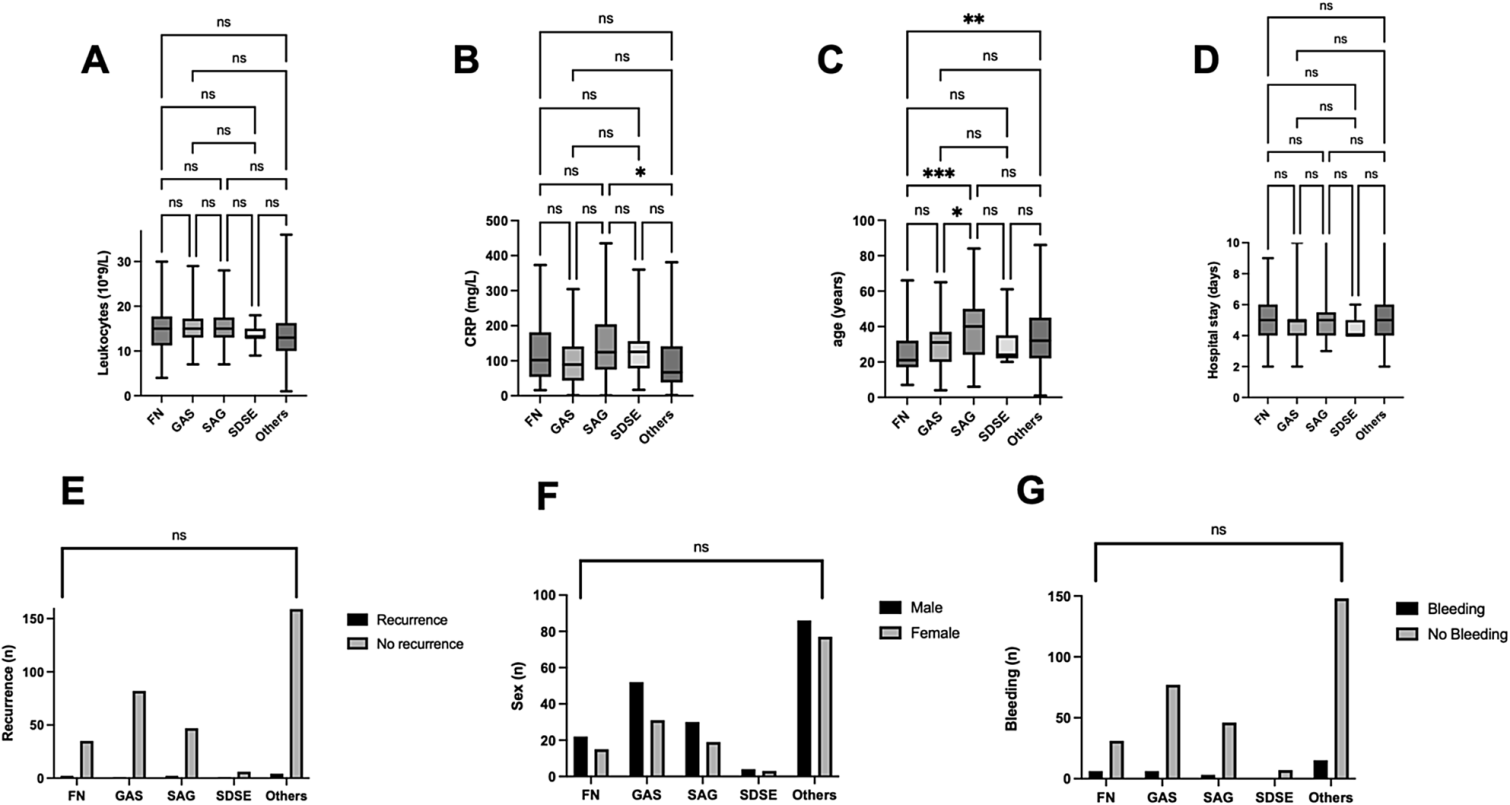
Characteristics of patients with *Fusobacterium necrophorum*, *S. pyogenes*, *S. anginosus group*, *Streprococcus dysgalactiae subspecies equisimilis*, and others (patients in whom none of the four most relevant pathogens were detected). (**A**) Leucocyte count at admission, (**B**) CRP-level at admission, (**C**) age, (**D**) length of hospital stay, (**E**) recurrence; (**F**) sex, (**G**) bleeding. FN: Fusobacterium necrophorum; GAS: S. pyogenes; SAG: S. anginosus group; SDSE: Streptococcus dysgalactiae subspecies equisimilis; CRP: C-reactive protein; ns: not significant; *: significant.

Interestingly, the presence of *FN* did neither correlate with a longer hospital stay (adjusted *p* = 0.888), nor with higher rates of surgical or infection-related complications (adjusted *p* = 0.537, adjusted *p* = 0.900). Figure 3 illustrates differences between patients with the *FN*, *S. pyogenes*, *S. anginosus*, *S. dysgalactiae*, and “others” (none of the aforementioned) and their correlation with the outcome parameters. Patients infected with *FN* (25.4 ± 12.5 years) and *S. pyogenes* (29.8 ± 13.1 years) were significantly younger than patients with *S. anginosus* (39.8 ± 19.2 years; *p* < 0.001 and *p* = 0.04, respectively). Additionally, patients with *FN* were significantly younger than those in whom none of the four most relevant pathogens were detected (36.1 ± 18.4 years; *p* = 0.001). Patients with detection of *S. anginosus* had significantly higher CRP levels (141.7 ± 98.7 mg/l vs. 97.5 ± 83.4 mg/l, adjusted *p* = 0.012) but no higher rate of infection-related complications (*n* = 5 (10.2%) vs. 8 (4.9%), adjusted *p* = 0.350) compared to patients in whom none of the four most relevant pathogens were detected. Patients with *S. pyogenes* showed a trend towards significantly higher leucocyte counts (15.6 ± 4.2 × 10^3^/µl vs. 13.8 ± 5.1 × 10^3^/µl, adjusted *p* = 0.054) compared to patients in whom none of the four most relevant pathogens were detected. Otherwise, none of the investigated pathogens was associated with an increased risk of surgical or infection-related complications. Similarly, none of the pathogens was associated with an increased risk of recurrence (Table 2). There was no case of Lemierre’s syndrome.

## Discussion

The present study provides a thorough overview of the microbiological spectrum and its association with the clinical course and management of consecutive patients treated for PTA in a large academic institution over the course of 5.5 years: The most frequently detected organisms were *Streptococcus pyogenes*, *Streptococcus anginosus* and *Fusobacterium necrophorum (FN)*. PTA with detection of *FN* mostly affected young adults with a mean age of 25.4 ± 12.5 years. Interestingly, although *FN* has previously been shown to be often implicated in severe complications such as Lemierre’s syndrome^18^, in our large cohort the detection of *FN* was not associated with the complication rate. Lemierre’s syndrome was not observed in our study. In accordance with local standards and clinical guidelines, all patients with a peritonsillar abscess were initially treated in an inpatient setting. The majority underwent tonsillectomy, which was unsurprisingly associated with lower rates of recurrence.

The demographic distribution of patients, with a mean age of 34.1 years and nearly equal gender distribution, aligns with the known epidemiology of PTA as primarily affecting adolescents and young adults^20^. Additionally, the average hospital stay in this study was 5.2 days, which is in line with other studies that report a mean hospital stay ranging from 4 to 7 days depending on the severity of the abscess and the treatment method employed^21^. This study shows no significant difference in hospital stay or leukocyte count between children/adolescents and adults, though adults exhibited higher mean CRP levels at admission, which may reflect more severe inflammation in adult cases of PTA, possibly due to later medical consultation. These results are consistent with previous studies^10^. The most common treatment was tonsillectomy (80.1%), followed by surgical incision and drainage (18.1%). In Germany, tonsillectomy has long been the standard intervention for PTA, which explains the high rate in our cohort. Nevertheless, recent national recommendations increasingly support conservative drainage approaches, reflecting a shift towards less invasive management^22^.Tonsillectomy was associated with longer hospital stays and a higher risk of surgical complications compared to incision and drainage. This finding supports the notion that while tonsillectomy can be more definitive in preventing recurrences, it may be associated with increased morbidity in terms of possibly severe postoperative complications^23^. Despite the higher bleeding risk, tonsillectomy remains a favored option in recurrent or severe cases, as it reduces the likelihood of abscess recurrence, which was observed at a low rate (1.2%) in the tonsillectomy cohort.

The microbiological analysis revealed that *Streptococcus pyogenes* (25.9%), *Streptococcus anginosus* (15.3%), and *Fusobacterium necrophorum* (11.5%) were the most frequently identified pathogens. These findings align with their well-established roles in the etiology of tonsillitis and related complications, particularly in cases of PTA^10^. Our study confirms *Streptococcus pyogenes* being associated with PTA in adults rather than in children or adolescents^24^. Interestingly, our study found a higher prevalence of *Fusobacterium necrophorum* in adolescents, consistent with previous reports describing it as a common pathogen in peritonsillar abscesses and acute tonsillitis in adolescents and young adults. This pathogen has been linked to a higher frequency of severe throat infections and is considered a leading cause of Lemierre’s syndrome, which also predominantly affects adolescents and young adults^25–27^. Björk et al. even hypothesized a transient oropharyngeal colonisation of *Fusobacterium necrophorum* in adolescents and young adults with the potential to cause infection under certain circumstances^26^. An interesting observation in our study was the absence of Lemierre’s syndrome despite the detection of *Fusobacterium necrophorum* in 11.5% of the patient cohort. However, this finding should be interpreted in the context of contemporary antibiotic and surgical management of PTA. In adequately treated patients, peritonsillar abscess and Lemierre’s syndrome appear to represent largely separate clinical entities, and the risk of progression to Lemierre’s syndrome after timely antibiotic and surgical therapy is considered very low. This highlights not only the role of early antibiotic administration but also suggests that factors beyond the presence of *Fusobacterium necrophorum* — such as individual host responses and the timing of medical intervention—may influence the clinical course and the risk of developing life-threatening complications like Lemierre’s syndrome^16^.

The study also highlights the diversity of other bacteria isolated, including gram-negative organisms such as *Klebsiella spp.*, *Escherichia coli*, and *Pseudomonas aeruginosa*. The presence of these pathogens, which are not typically associated with PTA, may reflect the inclusion of patients with hospital-acquired infections or those with underlying conditions. Our study demonstrated higher inflammatory markers in patients with detection of *S. anginosus*, underscoring the clinical relevance of this pathogen although there was no statistical association to higher complication rates^10,16^.

Empiric antibiotic therapy was predominantly based on aminopenicillin with or without beta-lactamase inhibitors, with only 3.1% of cases requiring a change in antibiotics based on culture results. This finding suggests that initial empiric therapy was generally appropriate for most patients, also reflected by the nearly complete susceptibility rates to first-line antibiotics in our cohort. The addition of a β-lactamase inhibitor aims to provide coverage for the potential involvement of anaerobic species and is in line with current guideline recommendations, just as the use of clindamycin as a second-line agent in cases of penicillin allergy^28^. In this study, the mean overall duration of antibiotic therapy (including both intravenous and oral administration) was 8.5 ± 2.7 days, with the initial intravenous course lasting on average 5.2 ± 2.3 days. This is consistent with previous studies that generally reported a treatment duration of 7 to 14 days for PTA depending on the severity of the infection and the patient’s response to treatment. Clinical guidelines frequently suggest that the intravenous phase is typically shorter, around 5 days, before transitioning to oral antibiotics to complete the course, similar to the findings of this study^28^. There is limited evidence supporting the use of antibiotic therapy after tonsillectomy for PTA. Our study did not investigate the potential benefits of postoperative antibiotics. However, Knipping et al. found no significant advantage in using antibiotics after tonsillectomy for PTA with respect to laboratory values, pain levels, or complications. To determine whether postoperative antibiotic therapy offers any real benefit, further prospective, randomized controlled trials are required^29^.

Complications occurred in 15.9% of patients, with postoperative bleeding being the most frequent one (8.7%). The study identified patients with elevated CRP levels as being at higher risk for infection-related complications, consistent with prior findings^10,30^. These findings emphasize the importance of close monitoring as higher CRP levels may be used to evaluate the risk for complications. Additionally, in immunocompromised individuals, there is often a greater variety of both aerobic and anaerobic organisms due to the immune system’s reduced capacity to contain infections. Furthermore, there is a higher risk of encountering opportunistic pathogens, as the reduced immune surveillance allows for greater bacterial load and persistence, potentially enabling more species to coexist and proliferate^10,31^. However, in our study cohort, the percentage of immunocompromised patients was too small to draw firm statistical conclusions from it.

This study has several limitations that must be considered. Firstly, its single-center and retrospective design limits the generalizability of the results to broader populations, particularly those in different healthcare settings or with varying microbial environments. Conclusions regarding children should also be drawn with caution, given the small number of children under 14 years of age in our cohort. The length of hospitalization and high tonsillectomy rates also reflect local treatment standards and may limit the generalizability of the results to other healthcare settings. Secondly, while the study included a relatively large cohort, the number of cases does not allow to draw final conclusions regarding the association between *Fusobacterium necrophorum* and Lemierre’s syndrome. Additionally, antibiotic escalation decisions were made clinically in only 3.1% of cases, which limits the evaluation of different antibiotic regimens and their role in pathogen clearance or complication prevention. This restricts the ability to assess the comparative effectiveness of alternative therapeutic strategies. The study also did not account for clinical variables such as abscess size and patient condition, both of which are relevant when deciding on surgical interventions versus medical management. Furthermore, it is unclear whether antibiotic therapy was initiated prior to microbiological sampling. Another limitation is the focus exclusively on surgically treated patients, which may introduce selection bias. Consequently, it remains unclear whether non-surgical, empirical medical treatment alone may be sufficient for smaller abscesses. Analyses regarding immunosuppressed patients and incision and drainage cases were limited by small subgroup sizes. Lastly, complication and recurrence rates were low, and the study was therefore not sufficiently powered to draw strong conclusions on these outcomes. Therefore, the present results should be interpreted as hypothesis-generating rather than definitive, and larger, adequately powered studies with multivariable adjustment are required to clarify whether specific pathogens are independently associated with recurrence or infectious complications.

Nevertheless, this study contributes valuable data on the clinical and microbiological characteristics of PTA and reaffirms the role of polymicrobial infections in PTA. Our findings validate existing epidemiological patterns of microbiological findings and the most involved pathogens but did not find an association between *FN* and increased complication rates. Our study also suggests that higher CRP levels at admission are associated with an increased risk of complications in patients with PTA which may help to assess the risk of complicated clinical courses. We hereby support a more differentiated and evidence-based approach to the management of PTA.

## Data Availability

The datasets generated and analyzed during the current study are available from the corresponding author on reasonable request.
